# Improving Nutrition and Activity Behaviors Using Digital Technology and Tailored Feedback: Protocol for the Tailored Diet and Activity (ToDAy) Randomized Controlled Trial

**DOI:** 10.2196/12782

**Published:** 2019-02-25

**Authors:** Rhiannon E Halse, Charlene L Shoneye, Christina M Pollard, Jonine Jancey, Jane A Scott, Iain S Pratt, Satvinder S Dhaliwal, Richard Norman, Leon M Straker, Carol J Boushey, Edward J Delp, Fengqing Zhu, Amelia J Harray, Maria A Szybiak, Anne Finch, Joanne A McVeigh, Barbara Mullan, Clare E Collins, Syed Aqif Mukhtar, Kieran N Edwards, Janelle D Healy, Deborah A Kerr

**Affiliations:** 1 School of Public Health Curtin University Perth, Western Australia Australia; 2 East Metropolitan Health Service Perth, Western Australia Australia; 3 Cancer Council WA Perth, Western Australia Australia; 4 Health Psychology & Behavioural Medicine Research Group School of Psychology Curtin University Perth, Western Australia Australia; 5 School of Physiotherapy and Exercise Science Curtin University Perth, Western Australia Australia; 6 Cancer Epidemiology Program University of Hawaii Cancer Center Honolulu, HI United States; 7 Department of Nutrition Purdue University West Lafayette, IN United States; 8 School of Electrical and Computer Engineering Purdue University West Lafayette, IN United States; 9 School of Occupational Therapy, Speech Therapy & Social Work Curtin University Perth, Western Australia Australia; 10 Movement Physiology Laboratory University of Witwatersrand Johannesburg South Africa; 11 School of Health Sciences, Faculty of Health and Medicine University of Newcastle Callaghan, New South Wales Australia; 12 Priority Research Centre in Physical Activity and Nutrition University of Newcastle Callaghan, New South Wales Australia; 13 Curtin Institute of Computation Curtin University Perth, Western Australia Australia

**Keywords:** obesity, diet, physical activity, sedentary, digital behavioral interventions, health behavior, wearable activity monitor, mHealth, eHealth, mobile food record

## Abstract

**Background:**

Excess weight is a major risk factor for chronic diseases. In Australia, over 60% of adults are overweight or obese. The overconsumption of energy-dense nutrient-poor (EDNP) foods and low physical activity (PA) levels are key factors contributing to population obesity. New cost-effective approaches to improve population diet and PA behaviors are needed.

**Objective:**

This 1-year randomized controlled trial (6-month intervention and 6-month follow-up) aims to investigate whether a tailored intervention using mobile technology can improve diet and PA behaviors leading to weight loss in adults (aged 18-65 years) who are overweight or obese and recruited through a social marketing campaign (LiveLighter).

**Methods:**

All eligible participants will provide data on demographics and lifestyle behaviors online at baseline, 6 months, and 12 months. Using two-stage randomization, participants will be allocated into one of three conditions (n=200 per group): tailored feedback delivered via email at seven time points, informed by objective dietary (mobile food record app) and activity (wearable activity monitor) assessment; active control receiving no tailored feedback, but undergoing the same objective assessments as tailored feedback; and online control receiving no tailored feedback or objective assessments. Primary outcome measures at 6 and 12 months are changes in body mass, EDNP food and beverage consumption, and daily moderate-to-vigorous PA (measured via accelerometry). Secondary outcomes include change in fruit and vegetable consumption, daily sedentary behaviors, and cost effectiveness.

**Results:**

Enrolment commenced in August 2017. Primary outcomes at 12 months will be available for analysis from September 2019.

**Conclusions:**

Tailored email feedback provided to individuals may deliver a cost-effective strategy to overcome existing barriers to improving diet and PA. If found to be successful and cost effective, upscaling this intervention for inclusion in larger-scale interventions is highly feasible.

**Trial Registration:**

Australian New Zealand Clinical Trials Registry ACTRN12617000554369; https://www.anzctr.org.au /Trial/Registration/TrialReview.aspx?id=371325&isReview=true

**International Registered Report Identifier (IRRID):**

DERR1-10.2196/12782

## Introduction

### Background

Excess weight is a major risk factor for chronic disease. Recent data indicate that more than 63% of Australian adults are overweight or obese, with higher rates observed in men than in women (68% versus 55%) [1]. The five leading attributable risk factors for burden of disease in Australia are poor diet, high body mass index (BMI), tobacco smoking, high blood pressure, and insufficient physical activity (PA) [2]. Of these factors, diet and PA are recognized as key factors for achieving energy balance in the complex development of overweight and obesity [3]. The 2011-2012 National Nutrition Survey reported that just over half of the adults met the recommendations for two serves of fruit, and only 7% met the recommended intake of five serves of vegetables [4]. Furthermore, 35% of the daily energy intake consumed was from “discretionary foods” (foods considered to be of little nutritional value; often high in saturated fats, added sugar, and salt; and alcohol or “junk” foods) [4]. With respect to PA, in 2011-2012, just 40% of adults met the recommended 30 minutes of daily moderate-to-vigorous PA (MVPA), and only 19% of adults achieved the recommended 10,000 steps per day [5]. Equally concerning, given the link between sedentary behavior, chronic disease, and obesity, 30% of adults reported engaging in more than 5 hours of sedentary leisure activity each day [5].

### Interventions in Overweight and Obese Populations

Key components of effective nutrition and PA behavioral change interventions include self-monitoring, feedback on performance, and goal setting [6-11]. More recently, there has been a move towards digital interventions utilizing mobile technology (eg, mobile apps and short message service [SMS] messaging) to improve population reach, real-time data collection, and feedback delivery [12]. Cost efficiency is a major potential strength of such interventions, and the challenge of ensuring design and implementation is supported by strong theoretical constructs. Although a plethora of healthy eating and weight-loss apps have become available, many lack behavioral strategies in their design [13]. A qualitative review of effective technology-based weight-loss interventions identified five key features related to effectiveness: self-monitoring, positive feedback, social support, controlled program content, and individually tailored feedback [14].

Tailored nutrition and PA interventions have shown promise for behavioral change; nonetheless, the effect size has been small, and most interventions thus far lack objective measures of PA [15-18]. Typically, feedback on behavior change is taken from self-report questionnaires, limiting the scope and relevance of individual diet and PA feedback. With the rapid advances in digital technologies, alternative mediums for delivery of information are now possible, including the use of images and other visual elements [19]. Therefore, interventions incorporating digital features provide a platform to test this concept and address concerns raised about the lack of models to inform the design of digital behavioral interventions [20,21]. For instance, a 6-month tailored intervention using the mobile food record (mFR) app for dietary assessment and tailored feedback improved the diet of young adults [22]. Features such as usability and willingness to continue to use apps may contribute to greater engagement and motivation enhancement by participants [20]. To date, few digital interventions have addressed both diet and PA behaviors together in an overweight population [9,23,24]. A unique aspect of this study is the detailed assessment of dietary intake and PA behaviors to inform tailored feedback.

### Aim

This study will use mobile technologies to undertake detailed assessment of dietary intake and PA behaviors and use these data to formulate personalized tailored feedback for study participants. The overall aim of this 1-year randomized controlled trial (RCT) is to investigate whether a tailored intervention using mobile technology can improve diet and PA behaviors in adults with overweight or obesity, recruited through the LiveLighter social marketing campaign in Perth, Western Australia.

## Methods

### Study Design

**Figure 1:**
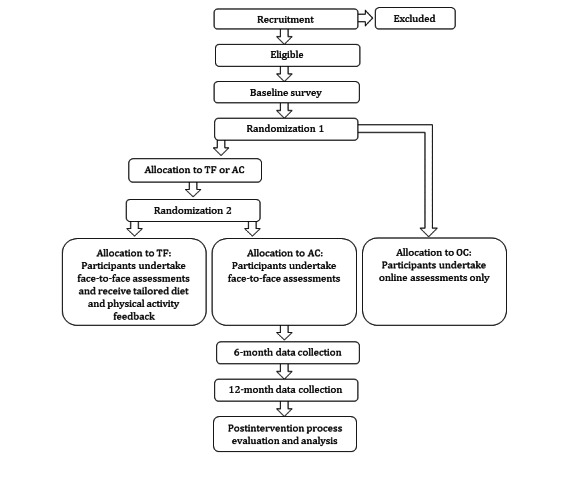
Study design with randomization to three groups. TF: tailored feedback; AC: active control; OC: online control.

This study is a 1-year RCT with a 6-month intervention and 6-month follow-up. Individuals who enroll via the LiveLighter website [25] will be invited to participate and, if eligible, will be randomized to one of three groups: (1) tailored feedback delivered via email at seven time points informed by objective dietary intake (mFR app) and activity (wearable activity monitor); (2) active control receiving no tailored feedback, but undergoing the same objective dietary and activity assessment as tailored feedback; and (3) online control receiving no tailored feedback or objective assessments (Figure 1). All groups will have access to publically available resources via the LiveLighter website. The inclusion of the online control group will distinguish monitoring and tailoring effects from those elicited by exposure to the LiveLighter social marketing campaign and website materials. The project protocol has been approved by the Curtin University Human Research Ethics Committee (approval number HR61/2016) and registered with the Australian New Zealand Clinical Trials Registry (ACTRN12617000554369).

### Recruitment

Participants in the Perth metropolitan area will be recruited using the LiveLighter website [25], LiveLighter social media campaigns, letter-box drops, and radio interviews directing interested individuals to further information and study registration on the LiveLighter website (Western Australia). Potential participants will complete an online consent and screening questionnaire. Staggered recruitment will take place over a 12-month period. To be eligible, participants must be aged 18-65 years, have a BMI ≥ 25 but <40 kg/m^2^, own a mobile telephone (iPhone or Android phone), be able to engage in regular PA, have internet access, and be available to visit a study center in metropolitan Perth. Participants will be excluded on the basis of serious illness or medical conditions including diabetes requiring insulin, renal disease, liver disease; weight loss > 4 kg in the previous 2 months; appetite suppressant use, weight loss, or hormone-replacement medication use; pregnancy or current breastfeeding; current tobacco smoking; daily alcohol consumption > 5 standard drinks; prior or planned weight loss surgery; and regular use of an activity monitor in the previous 12 months.

### Randomization

A two-staged block randomization will be used with allocation concealment from the active research team via the use of sealed opaque envelopes. The first randomization will be in blocks of six, with separate sex randomization. Second randomization will be in blocks of four, again with separate sex randomization. Eligible participants will be notified via email and invited to complete an online demographic and lifestyle behaviors questionnaire, as detailed in Table 1, prior to stage 1 randomization to either online control (n=200) or face-to-face (n=400) groups. Stage two randomization will occur at the second study visit to assign face-to-face participants to either tailored feedback or active control. Due to the nature of the intervention, it is not possible to blind participants or researchers to the intervention group postallocation. Sequence generation will be conducted by a biostatistician not involved in the implementation of the trial on site using a randomization table created in Stata (version 15, StataCorp, College Station, TX). The electronic file will be kept in a secure password-protected server by the statistician.

### Data-Collection Procedures

Following stage 1 randomization, tailored feedback and active control participants will be invited to attend two data-collection sessions with the research team, approximately 1 week apart. At the first baseline visit, participants will receive training in the use of the 4-day mFR [26-28] and hip-worn GT3X+ accelerometer (Actigraph, Pensacola, FL). During the second study visit, participants will return their accelerometer and be interviewed to clarify the content of their mFR images. At this visit, height, body mass, waist, and hip girth will be measured according to the standard protocol [29], and an aerobic fitness test (6-minute walk test) will be conducted [30]. The same assessments will be repeated at 6 and 12 months, along with additional online assessments for all groups (Table 1) at the same time points. All participants will have access to the LiveLighter website resources throughout the intervention and will be encouraged to use the materials that include evidence-based healthy recipes and meal plans [25].

### Research Study Database

A purpose-built research study database will be developed based on findings from prior research [44] using a Microsoft Access database platform to track the progress of the study participants at time points outlined in Tables 1 and 2. The database will have the functionality of sending autogenerated emails containing study information and links. To track progress of the tailored feedback group requiring face-to-face visits, information regarding upcoming appointment date and time and relevant survey URLs will be sent using autogenerated emails. To remind participants of upcoming appointments, email and mobile SMS prompts will be sent from the study database using “Email to SMS” technology.

An online survey tool (Qualtrics) will be used to capture demographic information as outlined in Table 1. The study database will have the functionality of importing data to automatically update participant status with respect to their study compliance. The system will prompt reminders via email and SMS for participants who have not yet completed their tasks.

### Dietary Assessment

For the face-to-face (active control and tailored feedback) groups, diet will be recorded using the mFR app with inclusion of a fiducial marker (an object of known shape, size, and color) [26] in the image to aid in portion size estimation. “Before eating” and “after eating” images of all foods and beverages consumed over four consecutive days, including one weekend day, will be captured at baseline, 6 months, and 12 months for each participant. In addition, at 6, 12, 18, and 24 weeks, the tailored feedback group will complete a 1-day mFR to encourage self-monitoring of food intake to facilitate feedback. All images will be automatically uploaded to a secure cloud server residing on the Curtin University Bentley campus via Wi-Fi or 3G/4G network. All images will be assessed by a research dietitian for serves of fruits, vegetables, and energy-dense nutrient-poor (EDNP) foods and beverages according to the Australian Guide to Healthy Eating standard serves (one serve=600 kJ) [45].

### Physical Activity Assessment

Physical activity and sedentary behavior will be assessed with a hip-worn Actigraph GT3X+ accelerometer to quantify change in average MVPA and sedentary time for the face-to-face (active control and tailored feedback) groups. The accelerometer will also enable the assessment of sleep. The participants will be instructed to wear the accelerometer on their right hip 24/7 for 7 consecutive days. Commonly used cut-off points will be used to classify each minute of accelerometer data as sedentary (<100 counts per minute) [46], light intensity (100-1951 counts per minute), moderate intensity (1952-5724 counts per minute), or vigorous intensity (>5724 counts per minute) [47].

A wrist-worn activity monitor (Fitbit Charge 2) will be provided to the tailored feedback group to enable 24/7 continuous self- and researcher monitoring of step count, MVPA, and hourly movement to inform PA feedback. Participants will be asked to wear the monitor at night to enable the assessment of sleep. With informed consent, activity monitor data will be automatically imported into a back-end research platform (Fitabase) to facilitate PA behavior data monitoring, extraction, and analysis by researchers.

### Intervention Content

Intervention content for diet and PA will be informed by evidence-based guidelines [48-50] to support weight loss through reduced energy intake and increased PA. The behavioral intervention technology framework Capability, Opportunity, Motivation, and Behaviour (COM-B) model; self-determination theory; and previous research will guide the development of intervention behavior-change strategies [20,22,51,52]. These include self-monitoring, goal setting, motivation enhancement (including positive reinforcement in tailoring communications and incentives), and feedback to increase likelihood of engagement [52]. To refine the intervention features and content, formative focus group studies with 56 consumers and health professionals were conducted prior to the intervention.

**Table 1:** Frequency of assessment of variables in the ToDAy study for the tailored feedback, active control, and online control groups.

Variables	Group	Baseline	6 months	12 months
Health status EQ-5D^a^, a 5-item scale to assess utility and health-related quality of life [31]	TF^b^, AC^c^, OC^d^	Yes	Yes	Yes
Height and body mass (self-report)	TF, AC, OC	Yes	Yes	Yes
Height, body mass, body mass index, waist, and hip girth^e^	TF, AC	Yes	Yes	Yes
Sociodemographics and personal characteristics assessed via questions on sex, age, eating behavior, educational level, country of birth, ethnicity, socioeconomic status, and financial status	TF, AC, OC	Yes	Yes	Yes
Australian eating survey, an online food frequency questionnaire with options for automated dietary feedback previously validated in adults [32]	TF, AC, OC	Yes	Yes	Yes
Dietary intake assessed by 4-day mFR^f^	TF, AC	Yes	Yes	Yes
Mobile food record usability to assess user feedback and method preference [33,34]	TF, AC	Yes	Yes	Yes
Three-factor eating survey to measure factors associated with eating behavior: cognitive restraint of eating, disinhibition, and hunger [35]	TF, AC	Yes	—^g^	—
Self-reported physical activity assessed via The International Physical Activity Questionnaire (short form) [36]	TF, AC, OC	Yes	Yes	Yes
Cardiorespiratory fitness determined by distance covered in the 6-minute walk test to assess change in submaximal exercise capacity [30]	TF, AC	Yes	Yes	Yes
Physical activity and sedentary behavior assessed with GT3X+ Actigraph accelerometer to quantify change in average minutes of moderate-to-vigorous physical activity and sedentary time	TF, AC	Yes	Yes	Yes
Sleep-quality assessment using the Pittsburgh Sleep Quality Index seven-component evaluation of sleep quality, latency, duration, habitual sleep efficiency, sleep disturbances, use of sleeping medications, and daytime dysfunction over the previous month [37]	TF, AC	—	Yes	Yes
Depression, anxiety, stress scale, with 21 self-report items to assess severity of depression, anxiety, and stress [38]	TF, AC, OC	Yes	Yes	Yes
Fear of negative evaluation, with twelve 5-point items to assess concern about being perceived unfavorably [39]	TF, AC	Yes	—	—
Social desirability to measure social approval and acceptance [40]	TF, AC	Yes	—	—
Habit Index Score [41]	TF, AC	Yes	Yes	Yes
Weight-loss history, an 8-item tool to assess previous weight-loss history [42]	TF, AC, OC	—	—	—
Technology use questionnaire to assess duration and frequency of technology use indicative of sedentary behaviors [43]	TF, AC, OC	Yes	Yes	Yes
Feedback evaluation questionnaire for activity-monitor usability, physical activity, and dietary feedback evaluation	TF	—	Yes	Yes

^a^EQ-5D: EuroQol-5D.

^b^TF: tailored feedback.

^c^AC: active control.

^d^OC: online control.

^e^Height measured in centimeters via stadiometer using stretch stature method, body mass measured in kilograms via weighing scale in minimal clothing at similar time of the day, body mass index calculated as kilogram per meter squared, waist measured in in centimeters via tape at the narrowest point between the lower costal border and iliac crest, and hip girth measured via tape at the level of the greatest posterior protuberance.

^f^mFR: mobile food record.

^g^Not assessed.

**Table 2:** Overview of frequency, content, and technique of tailored email feedback for diet and physical activity behaviors.

Feedback frequency	Feedback content		Behavior-change techniques [52]
	Diet	Physical activity	
2 weeks	4-d summary (average + range) from mFR^a^ showing average daily serves (kilojoule equivalent) for EDNP^b^ foods, sugary drinks, and alcohol Example ADG^c^ serves for EDNP foods, sugary drinks, and alcohol mFR food images showing participants the source of their EDNP foods, sugary drinks, and alcohol serves Tailored feedback + goals based on key messages: avoid EDNP foods, avoid sugary drinks, avoid alcohol	Introduction of movement goals and wearable activity monitor guide 7-d summary (average + range) for step count, MVPA^d^, and hourly movement Tailored feedback + guidance based on movement goal achievement (not met, almost met, and met)	Instruction on how to perform the behavior Feedback on behavior Tailored personalized message Prompt self-monitoring of behavior Goal setting Discrepancy between current behavior and recommendations
4 weeks	Weight loss goal of 10% 4-d summary (average + range) from mFR showing average daily serves for fruit and vegetables Example ADG serves for fruit and vegetables mFR food images showing participants the source of their fruit and vegetable intake Tailored feedback against recommended serves + goals to increase fruit and vegetable serves	7-d visual summary of challenge movement goal, with comparison to goal target Activity tips to assist with weight loss linked to movement goals	Review behavior goal Feedback on behavior Action planning Prompt self-monitoring of behavior Discrepancy between current behavior and recommendations
6 weeks	1-d summary from 6-wk mFR showing daily serves of EDNP foods, sugary drinks, alcohol, and fruits and vegetables Comparison against baseline diet mFR food images showing participants the source of their EDNP foods, sugary drinks, alcohol, fruits, and vegetables Tailored feedback against recommended serves + goals targeting: Avoid or limit EDNP foods, sugary drinks and alcohol Eat less at meals or snacks (except for fruit and vegetables) Eat less often (eg, limit snacking)	Review of the movement goals 7-d summary (average + range) for step count, MVPA, and hourly movement Comparison with baseline activity for each movement goal Tailored feedback + guidance based on movement goal achievement (not met, almost met, and met)	Review behavior goals Self-comparison Feedback on behavior Tailored personalized message Prompt self-monitoring of behavior Goal setting Discrepancy between current behavior and recommendations
12 weeks	1-d summary from 12-wk mFR showing daily serves of EDNP foods, sugary drinks, alcohol mFR food images showing participants the examples of meal or snack behaviors Tailored feedback + goals targeting: Eat less at meals or snacks (except for fruits and vegetables) Avoid or limit snacking	Reiteration of energy deficit goal + instruction on how to create an energy deficit via energy output 7-d summary (average + range) of MVPA goal Tailored feedback + guidance based on movement goal achievement (not met, almost met, and met) Translation of MVPA into energy output	Review of outcome goal Review behavior goal Feedback on behavior Tailored personalized message Prompt self-monitoring of behavior Goal setting Discrepancy between current behavior and recommendations
18 weeks	Reiteration of dietary goals + instruction on how to create an energy deficit from diet (eg, reduction in EDNP food serves) Reiteration of “what’s a serve of EDNP foods” Reminder of dietary goals + suggestions on how to achieve them: Avoid or limit EDNP foods, sugary drinks, and alcohol Eat less at meals or snacks (except for fruit and vegetables) Eat less often (eg, limit snacking)	Reiteration of energy-deficit goal + instruction on how to create an energy deficit via energy output 7-d summary (average + range) of MVPA goal Tailored feedback + guidance based on movement goal achievement (not met, almost met, and met) Translation of MVPA into energy output Reminder of movement goal targets linked to creating an energy deficit	Review of outcome goal Review behavior goals Feedback on behavior Tailored personalized message Goal setting Discrepancy between current behavior and recommendations
6 months	4-d summary from 6-mo mFR showing daily serves with comparison against baseline diet mFR food images showing participants the source of their EDNP foods, sugary drinks, alcohol, fruits, and vegetables Tailored feedback against recommended serves + goals targeting: Avoid or limit EDNP foods, sugary drinks, and alcohol Eat less at meals or snacks (except for fruit and vegetables) Eat less often (eg, limit snacking) Tailored support for unhelpful behaviors: Emotional/restrained/uncontrolled eating (identified in the three-factor eating questionnaire) Tailored feedback on how to make a healthy diet habitual based on habit index score	Face-to-face visit summary: comparison with baseline (body mass and aerobic fitness) 7-d visual summary (average) for step count, MVPA, and hourly movement + comparison with baseline for each movement goal Future goal setting for translation phase Tailored guidance on how to make PA habitual based on habit index score	Review behavior goals Self-comparison Feedback on behavior Tailored personalized message Prompt self-monitoring of behavior Goal setting Discrepancy between current behavior and recommendations
12 months	4-d summary from 12-mo mFR showing daily serves with comparison against baseline Tailored feedback against recommended serves + target goals	Face-to-face visit tabulated summary: comparison with baseline and 6 mo (body mass and aerobic fitness) 7-d visual summary (average) for step count, MVPA, and hourly movement + comparison with baseline and 6 mo for each movement goal Future goal setting for translation phase	Review behavior goals Self-comparison Social comparison with study participants Feedback on behavior Tailored personalized message Prompt self-monitoring of behavior Goal setting Discrepancy between current behavior and recommendations

^a^mFR: mobile food record.

^b^EDNP: energy-dense nutrient poor.

^c^ADG^:^ Australian Dietary Guidelines.

^d^MVPA: moderate-to-vigorous physical activity.

At randomization, tailored feedback participants will be informed of feedback email frequency and content and that they may opt out of correspondence at any time by informing the research team. Email templates will be developed for each of the seven time points, containing personalized dietary and PA feedback content for each participant. Feedback will be consistent with communications from the LiveLighter campaign, Australian Dietary Guidelines, and the Australian PA and Sedentary Behavior Guidelines [48-50]. The content will address each participant’s personal barriers to changing key diet and PA behaviors, reinforce motivation, and guide the adoption of health-enhancing habits. Tailored feedback on diet and PA behaviors will commence within 2 weeks of baseline and continue at 4, 6, 12, 18 weeks and 6 and 12 months thereafter. The feedback emails will be sent from Monday to Friday during business hours (9 AM to 5 PM). Components used in tailoring will include self-comparison, preference for autonomy support, intention, motivation, confidence informed by self-determination theory, and motivational interviewing (Table 2) [52,53].

### Message Tailoring

Tailoring involves creating communications in which information about an individual is used to determine specific content he or she receives [15,54]. Positive effects of tailoring have been demonstrated in changing diet and PA behaviors [15-17,55]. The intention of tailoring, which uses characteristics unique to the individual, is to improve behavioral outcomes by altering processing or making the message more acceptable [54,56]. These characteristics can include personal behaviors, psychosocial characteristics, and dietary and PA behaviors. Specific strategies for message tailoring include personalization, feedback, and content matching [54]. This study will focus on personalized feedback (exemplified in Table 3) using information obtained on diet and PA behaviors at baseline and specific time points throughout the intervention. Digital elements (food and beverage images and graphical presentation of PA data) will be included to enable evaluation of these components.

### Tailored Dietary Feedback

Tailored dietary feedback will be formulated by the research dietitian based on food group analysis of the 4-day mFR. Feedback will focus on key messages encouraging daily energy reduction of 2000 kJ by avoiding or limiting EDNP foods, sugar-sweetened beverages, and alcohol; eating less at meals or additional snacks (except for salad and vegetables); and eating less often. Food group serves will be categorized for each participant based on three defined target zones (not achieved, almost achieved, and achieved). A template will be used for each dietary feedback email, modified according to the results of each participants’ dietary analysis. For EDNP serves, the template will be modified according to dietary intake to indicate average daily serves of “junk” foods, sugary drinks, and alcohol and kilojoule intake. Participants will be shown an image of their dietary sources of EDNP food and beverages. For fruit and vegetable serves, a scripted message will be devised for three levels of intake: (1) low: 0 to <3.5 serves of fruits and vegetables, (2) medium: 3.5 to <7 serves of fruits and vegetables, and (3) meeting the recommendation: at least 2 serves of fruits and 5 serves of vegetables per day. Individual mFR images will be incorporated into email templates to illustrate the sources of EDNP foods, fruits, and vegetable serves. Two to three suggested modifications will be provided to each participant to support them in achieving the daily energy-reduction goal.

### Tailored Activity Feedback

Individual activity data will inform PA feedback based on activity monitor recordings 1 week prior to the feedback time point. The data will be automatically imported into a research platform (Fitabase) to facilitate monitoring and analysis of continuous back-end data. Messages will focus on the three movement goals: “move more” (step count; toward ≥10,000 steps), “move harder” (minutes spent in MVPA; towards ≥30 active minutes), and “move more often” (hourly movement; towards ≥250 steps per hour). Participants will receive tailored email feedback regarding their current activity, and guidance on goal progression to classify goal achievement (not achieved, almost achieved, or achieved).

### Control Groups

The online control group will complete online self-report questionnaires only, while the active control group will also undertake face-to-face data collection and record dietary intake (mFR app) and PA behaviors (accelerometery) at baseline, 6 months, and 12 months. Neither group will receive tailored messaging or feedback on their dietary intake or PA. As an incentive for retention, active control participants will be advised that they will receive feedback upon study completion, and the online control group will be entered into a 6-monthly prize draw to encourage ongoing participation.

### Economic Evaluation

An economic evaluation will be conducted to consider the relative costs and outcomes of the intervention. To facilitate a cost-utility analysis, the EuroQol-5D will be administered. This is a widely used instrument specifically designed to capture quality of life for health economics [57]. This study will use the five-level version of the instrument to identify sensitive and small, but important, changes in health-related quality of life [58]. Quality-adjusted life years will be estimated for intervention and control groups. Concerning costs, we will collect the time needed to provide tailored advice to participants, medication and supplement use (name, dose, and frequency), and family expenditure on groceries. This will allow economic evaluation from the perspective of the health system (by considering only the cost of the intervention and medication) and a broader society (by considering all costs). Univariate and multivariate sensitivity analyses will be undertaken. In particular, the impact of different methods of extrapolating costs and outcomes beyond the horizon of the trial will be assessed. The costing model will include resources required to assess ongoing maintenance of the mFR and wearable activity monitors, including changes as a result of upgrades to operating systems. We will also record minor costs of ongoing use (SMS messages and email communication). The major cost of the intervention is likely to be provision of tailored advice based on data received (research personnel and research platform costs). This will be estimated by recording time spent deconstructing the mobile app data, the Web app, and objective measures and then interpreting data and constructing appropriate feedback.

**Table 3:** Examples of tailored feedback intervention strategies.

Type^a^	Example	Processing and goals
Descriptive (what is known)	Hi Jane, it’s the team with your feedback. So how did you score? Ave fruit serves = 1.5, ave veg serves = 3. What’s the goal again? 2 fruit & 5 veg every day. You are halfway there!	Effortful processing and self-referencing
Comparative (contrasts with others)	Hi Jane, so how did you score? Ave fruit serves = 1.5, veg serves = 3.5. Your fruit serves varied from 0 - 3.5, veg from 1.5 - 5.5 over 4 days. So how does your intake compared to others? Ave fruit serves = 1, veg serves = 2.	Effortful processing, self-referencing, and normative beliefs and attitudes
Evaluative (interpretation)	Hi Jane, it’s the team with feedback on your PA. So how did you score? Ave steps a day = 5,500. What’s your goal again? 10,000 steps a day. You are over halfway there!	Effortful processing, self-referencing, and normative beliefs and attitudes

^a^Adapted from [54].

### Process Evaluation

Process evaluation will assess to what extent the intervention reached the target audiences. A brief questionnaire will be used to evaluate participants’ perception of the tailored feedback (ie, message persuasiveness, message tone, readability, ease of understanding, usefulness of advice, suitability, and relevance to age group) and to comment on features they like/dislike about the program. Usability feedback will also be obtained for the mFR (tailored feedback and active control groups) and activity monitor (tailored feedback group only). Impact evaluation will consist of exit interview surveys via telephone at 14 months to assess intervention impact as well as perceptions of various strategies and materials. Selected program completers and noncompleters (tailored feedback and active control groups) will be invited to participate in one-on-one interviews concerning their perceptions of the ToDAy intervention.

### Statistical Analysis

The following primary outcome variables will be measured at baseline and 12 months of the intervention: changes in body mass, EDNP food and beverage consumption (sugar-sweetened beverages, alcohol, and take away and other “junk” foods), and daily minutes spent in MVPA. Secondary outcome variables include changes in fruit and vegetable serves and daily minutes spent in sedentary activity. Outcomes will identify characteristics of responders who remained engaged and reported improvements in key behaviors and quantify resource use associated with each arm of the study, which will be used in an economic evaluation of tailored feedback. Data on change in outcome variables in each of the two groups will be compared using analysis of covariance. Assumptions of the analyses will be assessed by examining residuals. Data will be transformed if assumptions of the analyses are not satisfied. Possible covariates considered will include age, sex, country of birth, ethnicity, highest education level, socioeconomic index for area, and baseline value of the variable analyzed. *P*-values < .05 will be considered statistically significant. Effect size of differences between treatment and control will be expressed as adjusted mean difference and associated 95% CIs. Data on change in outcome variables postintervention and follow-up time points will be converted into binary categorical variables and analyzed using multivariable logistic regression and generalized estimating equations. Odds ratio and associated 95% CIs will be reported. The analyses will identify characteristics of participants who are least likely to change their consumption of EDNP foods, PA, and BMI, thereby identifying target groups for future health promotion interventions.

### Power

A sample size of 600 participants (n=200 per group) will have sufficient power to detect a change in the primary outcome variable of at least 0.6 median serves/day of EDNP (discretionary) foods (or equivalent to a 360 kJ/day reduction) between groups at 90% power and 5% level of significance. Assuming a drop-out rate of 20%, a total of 600 participants will be recruited.

## Results

Enrollment commenced in August 2017. Primary outcomes at 12 months will be available for analysis from September 2019.

## Discussion

### Overview

Improving participation and reach is a challenge for population-based obesity interventions. Worldwide participation rates in population studies are declining [59], and while enrollment in studies involving face-to-face recruitment is somewhat higher [59], there is a need to evaluate the effectiveness of digital interventions that include multiple strategies to improve engagement [60]. Interventions utilizing digital technologies may have greater appeal in overweight and obese populations, as participants may feel more comfortable completing self-reported questionnaires in a more anonymous setting [61].

Reasons for the lack of adherence to lifestyle recommendations are poorly understood. One contributing dietary factor may be that many adults incorrectly believe their diet to be healthy when it is not [62]. This mismatch between perceived and actual intake may be corrected by more accurate, objective dietary assessment; comparison with national recommendations; and clear, relatable tailored feedback. Furthermore, despite sustained public health efforts, the majority of Australian adults are “inactive,” failing to meet the recommended PA guidelines [63]. Thus, focus on the most-effective messaging strategy (both content and delivery) to target these behaviors is needed. A systematic review of tailored interventions and the identified lack of objective measurements in studies on dietary and PA behaviors are key limitations of tailored interventions [17]. This, in part, is due to difficulty in undertaking more objective methods of dietary and PA assessment on a large scale. Exploring digital methods of assessment may allow more cost-effective approaches to be implemented.

Tailoring focuses on characteristics unique to the person with the intention of improving behavioral outcomes by making the message more acceptable to each individual. Characteristics of messages and feedback that can be tailored include personal behaviors, psychosocial characteristics, and diet and PA behaviors [54]. Personal relevance is key, and dynamic (ongoing assessment) tailoring versus static tailoring (one baseline assessment) has stronger effects over time [15]. Effective tailoring strategies include multiple intervention contacts and iterative feedback [19]. Of note, tailoring may be more effective for men than women [64].

Numerous behavioral theories have been used as a basis for tailored interventions, including the Stages of Change Model and Precaution Adoption Process Model [19]. However, there is increasing support for self-determination theory in weight control, diet, and tailored PA interventions to address autonomous motivation and self-regulation [52,53,65-68]. Central to the self-determination theory is an emphasis on autonomous behaviors (originating from one’s-self), as opposed to pressure or coercion into a particular course of action when delivering advice [67]. This provides a framework for the style of communication to be used in tailored interventions but does not address approaches used in digital technology interventions. Researchers have raised concerns about the lack of models to inform design of technology-based behavioral interventions [20,21]. In response, Mohr et al [20] proposed a behavioral intervention technology framework for interventions that use a range of technologies, including mobile phones, the Web, and sensors aimed at changing behavior. Features such as usability and willingness to continue to use the app may contribute to enhanced participant engagement and motivation [20]. Michie et al [68] developed the COM-B model, which guides researchers to identify behavioral targets and subsequent psychological theories for behavior-change interventions. The COM-B model identifies capability, opportunity, and motivation as the three core categories to perform a behavior. This means that to perform a behavior, individuals must be capable with physical and mental ability (eg, nutrition knowledge and cooking skills) as well as practical and social opportunities (eg, access to affordable and healthy food). Motivation includes automatic drivers like habits as well as goals, beliefs, plans, and impulses. Assessing these determinants is the first step to identify interventions and theories that can help change behavior.

With respect to PA, wearable technology is being rapidly adopted, with over one-third of the Australian population using activity monitors to record PA and sedentary behaviors [69]. These devices are becoming vital in the context of research to facilitate monitoring of activity in real-time and under free-living conditions. Nonetheless, a gap exists between recording/self-monitoring and behavioral change. Furthermore, there is a lack of strongly designed studies that have considered the combination of behavioral theory with activity monitors to improve health behaviors. New-generation activity monitors allow for critical information to be harnessed from large-scale research studies. In this study, activity monitors will be used to record 24/7 behavior, specifically active and sedentary minutes; exercise intensity; nonsedentary hours; and step count in overweight adults. Together with dietary analysis, curation of these data will enable provision of detailed, richer feedback to participants and may therefore be more effective in helping change diet and PA behaviors. If found successful, the approach used in this study could be incorporated into larger-scale health campaigns.

### Conclusions

The current obesity epidemic is occurring against a background of a decline in PA participation and increasingly poor dietary choices, with the incidence of both obesity and prevalence of inactivity worsening with age. Promoting and maintaining healthy diet and PA behaviors through personalized tailored feedback is feasible, novel, and potentially cost effective. Personalized feedback with comparison to recommendations and guidance in forming healthy habits is essential to overcome existing barriers to lifestyle change. Digital technologies (mobile apps, email, and web) have the potential to reach larger populations of healthy adults and those at risk of chronic disease but to date have not been fully explored. The outcomes of this intervention may have the potential to positively impact health at a boarder population level, with findings informing translation of “best practice” lifestyle intervention aimed at overweight adults.

## Abbreviations

BMI: body mass index
COM-B: Capability, Opportunity, Motivation, and Behaviour
EDNP: energy-dense nutrient-poor foods
mFR: mobile food record
MVPA: moderate-to-vigorous physical activity
OC: online control
PA: physical activity
RCT: randomized controlled trial
SMS: short message service
TF: tailored feedback

## References

[1] (2011). Profiles of Health, Australia, 2011-13.

[2] (2010). Institute for Health Metrics and Evaluation.

[3] Australian Government Department of Health, National Health and Medical Research Council (2013). Clinical Practice Guidelines for the Management of Overweight and Obesity for Adults, Adolescents and Children in Australia.

[4] (2011). Australian Bureau of Statistics.

[5] (2011). Australian Bureau of Statistics.

[6] Michie S, Abraham C, Whittington C, McAteer J, Gupta S (2009). Effective techniques in healthy eating and physical activity interventions: a meta-regression. Health Psychol.

[7] Straker LM, Howie EK, Smith KL, Fenner AA, Kerr DA, Olds TS, Abbott RA, Smith AJ (2014). The impact of Curtin University's activity, food and attitudes program on physical activity, sedentary time and fruit, vegetable and junk food consumption among overweight and obese adolescents: a waitlist controlled trial. PLoS One.

[8] Morgan PJ, Lubans DR, Collins CE, Warren JM, Callister R (2009). The SHED-IT randomized controlled trial: evaluation of an Internet-based weight-loss program for men. Obesity (Silver Spring).

[9] Turk MW, Elci OU, Wang J, Sereika SM, Ewing LJ, Acharya SD, Glanz K, Burke LE (2013). Self-monitoring as a mediator of weight loss in the SMART randomized clinical trial. Int J Behav Med.

[10] Eckerstorfer LV, Tanzer NK, Vogrincic-Haselbacher C, Kedia G, Brohmer H, Dinslaken I, Corcoran K (2018). Key Elements of mHealth Interventions to Successfully Increase Physical Activity: Meta-Regression. JMIR Mhealth Uhealth.

[11] Burke L, Lee AH, Pasalich M, Jancey J, Kerr D, Howat P (2012). Effects of a physical activity and nutrition program for seniors on body mass index and waist-to-hip ratio: a randomised controlled trial. Prev Med.

[12] Michie S, Yardley L, West R, Patrick K, Greaves F (2017). Developing and Evaluating Digital Interventions to Promote Behavior Change in Health and Health Care: Recommendations Resulting From an International Workshop. J Med Internet Res.

[13] Pagoto S, Schneider K, Jojic M, DeBiasse M, Mann D (2013). Evidence-based strategies in weight-loss mobile apps. Am J Prev Med.

[14] Khaylis A, Yiaslas T, Bergstrom J, Gore-Felton C (2010). A review of efficacious technology-based weight-loss interventions: five key components. Telemed J E Health.

[15] Krebs P, Prochaska JO, Rossi JS (2010). A meta-analysis of computer-tailored interventions for health behavior change. Prev Med.

[16] Kroeze W, Werkman A, Brug J (2006). A systematic review of randomized trials on the effectiveness of computer-tailored education on physical activity and dietary behaviors. Ann Behav Med.

[17] Broekhuizen K, Kroeze W, van Poppel MNM, Oenema A, Brug J (2012). A systematic review of randomized controlled trials on the effectiveness of computer-tailored physical activity and dietary behavior promotion programs: an update. Ann Behav Med.

[18] Short CE, James EL, Plotnikoff RC, Girgis A (2011). Efficacy of tailored-print interventions to promote physical activity: a systematic review of randomised trials. Int J Behav Nutr Phys Act.

[19] Noar SM, Benac CN, Harris MS (2007). Does tailoring matter? Meta-analytic review of tailored print health behavior change interventions. Psychol Bull.

[20] Mohr DC, Schueller SM, Montague E, Burns MN, Rashidi P (2014). The behavioral intervention technology model: an integrated conceptual and technological framework for eHealth and mHealth interventions. J Med Internet Res.

[21] Riley WT, Rivera DE, Atienza AA, Nilsen W, Allison SM, Mermelstein R (2011). Health behavior models in the age of mobile interventions: are our theories up to the task?. Transl Behav Med.

[22] Kerr DA, Harray AJ, Pollard CM, Dhaliwal SS, Delp EJ, Howat PA, Pickering MR, Ahmad Z, Meng X, Pratt IS, Wright JL, Kerr KR, Boushey CJ (2016). The connecting health and technology study: a 6-month randomized controlled trial to improve nutrition behaviours using a mobile food record and text messaging support in young adults. Int J Behav Nutr Phys Act.

[23] Wang J, Cai C, Padhye N, Orlander P, Zare M (2018). A Behavioral Lifestyle Intervention Enhanced With Multiple-Behavior Self-Monitoring Using Mobile and Connected Tools for Underserved Individuals With Type 2 Diabetes and Comorbid Overweight or Obesity: Pilot Comparative Effectiveness Trial. JMIR Mhealth Uhealth.

[24] Jakicic JM, Davis KK, Rogers RJ, King WC, Marcus MD, Helsel D, Rickman AD, Wahed AS, Belle SH (2016). Effect of Wearable Technology Combined With a Lifestyle Intervention on Long-term Weight Loss: The IDEA Randomized Clinical Trial. JAMA.

[25] (2018). LiveLighter.

[26] Zhu F, Bosch M, Woo I, Kim S, Boushey CJ, Ebert DS, Delp EJ (2010). The Use of Mobile Devices in Aiding Dietary Assessment and Evaluation. IEEE J Sel Top Signal Process.

[27] Zhu F, Bosch M, Khanna N, Boushey CJ, Delp EJ (2015). Multiple hypotheses image segmentation and classification with application to dietary assessment. IEEE J Biomed Health Inform.

[28] Ahmad Z, Kerr DA, Bosch M, Boushey CJ, Delp EJ, Khanna N, Zhu F (2016). A Mobile Food Record For Integrated Dietary Assessment. MADiMa16 (2016).

[29] Stewart A, Marfell-Jones M, Olds T, de Ridder JH (2011). International standards for anthropometric assessment.

[30] Rikli RE (2000). Reliability, validity, and methodological issues in assessing physical activity in older adults. Res Q Exerc Sport.

[31] Rabin R, de Charro F (2001). EQ-5D: a measure of health status from the EuroQol Group. Ann Med.

[32] Collins CE, Boggess MM, Watson JF, Guest M, Duncanson K, Pezdirc K, Rollo M, Hutchesson MJ, Burrows TL (2014). Reproducibility and comparative validity of a food frequency questionnaire for Australian adults. Clin Nutr.

[33] Boushey CJ, Harray AJ, Kerr DA, Schap TE, Paterson S, Aflague T, Bosch RM, Ahmad Z, Delp EJ (2015). How willing are adolescents to record their dietary intake? The mobile food record. JMIR Mhealth Uhealth.

[34] Kerr DA, Dhaliwal SS, Pollard CM, Norman R, Wright JL, Harray AJ, Shoneye CL, Solah VA, Hunt WJ, Zhu F, Delp EJ, Boushey CJ (2017). BMI is Associated with the Willingness to Record Diet with a Mobile Food Record among Adults Participating in Dietary Interventions. Nutrients.

[35] Stunkard AJ, Messick S (1985). The three-factor eating questionnaire to measure dietary restraint, disinhibition and hunger. J Psychosom Res.

[36] Craig CL, Marshall AL, Sjöström M, Bauman AE, Booth ML, Ainsworth BE, Pratt M, Ekelund U, Yngve A, Sallis JF, Oja P (2003). International physical activity questionnaire: 12-country reliability and validity. Med Sci Sports Exerc.

[37] Backhaus J, Junghanns K, Broocks A, Riemann D, Hohagen F (2002). Test-retest reliability and validity of the Pittsburgh Sleep Quality Index in primary insomnia. J Psychosom Res.

[38] Sinclair SJ, Siefert CJ, Slavin-Mulford JM, Stein MB, Renna M, Blais MA (2012). Psychometric evaluation and normative data for the depression, anxiety, and stress scales-21 (DASS-21) in a nonclinical sample of U.S. adults. Eval Health Prof.

[39] Tooze JA, Subar AF, Thompson FE, Troiano R, Schatzkin A, Kipnis V (2004). Psychosocial predictors of energy underreporting in a large doubly labeled water study. Am J Clin Nutr.

[40] Marlowe D, Crowne DP (1961). Social desirability and response to perceived situational demands. J Consult Psychol.

[41] Allom Vanessa, Mullan Barbara, Cowie Eloise, Hamilton Kyra (2016). Physical Activity and Transitioning to College: The Importance of Intentions and Habits. Am J Health Behav.

[42] Myers VH, McVay MA, Champagne CM, Hollis JF, Coughlin JW, Funk KL, Gullion CM, Jerome GJ, Loria CM, Samuel-Hodge CD, Stevens VJ, Svetkey LP, Brantley PJ (2013). Weight loss history as a predictor of weight loss: results from Phase I of the weight loss maintenance trial. J Behav Med.

[43] Hands BP, Chivers PT, Parker HE, Beilin L, Kendall G, Larkin D (2011). The associations between physical activity, screen time and weight from 6 to 14 yrs: the Raine Study. J Sci Med Sport.

[44] Harray AJ, Boushey CJ, Pollard CM, Delp EJ, Ahmad Z, Dhaliwal SS, Mukhtar SA, Kerr DA (2015). A Novel Dietary Assessment Method to Measure a Healthy and Sustainable Diet Using the Mobile Food Record: Protocol and Methodology. Nutrients.

[45] Australian Government - Department of Health and Aging, National Health and Medical Research Council (2013). Eat for Health - Educator Guide: Information for nutrition educators.

[46] Matthews CE, Chen KY, Freedson PS, Buchowski MS, Beech BM, Pate RR, Troiano RP (2008). Amount of time spent in sedentary behaviors in the United States, 2003-2004. Am J Epidemiol.

[47] Freedson PS, Melanson E, Sirard J (1998). Calibration of the Computer Science and Applications, Inc. accelerometer. Med Sci Sports Exerc.

[48] Australian Government Department of Health (2017). Australia's Physical Activity and Sedentary Behavior Guidelines for Adults (18-64 years).

[49] (2018). Physical Activity Guidelines for Americans, 2nd edition.

[50] Australian Government - Department of Health and Aging, National Health and Medical Research Council (2013). Australian dietary guidelines - providing the scientific evidence for healthier Australian diets.

[51] Pollard CM, Howat PA, Pratt IS, Boushey CJ, Delp EJ, Kerr DA (2016). Preferred Tone of Nutrition Text Messages for Young Adults: Focus Group Testing. JMIR Mhealth Uhealth.

[52] Michie S, Richardson M, Johnston M, Abraham C, Francis J, Hardeman W, Eccles MP, Cane J, Wood CE (2013). The behavior change technique taxonomy (v1) of 93 hierarchically clustered techniques: building an international consensus for the reporting of behavior change interventions. Ann Behav Med.

[53] Resnicow K, Davis RE, Zhang G, Konkel J, Strecher VJ, Shaikh AR, Tolsma D, Calvi J, Alexander G, Anderson JP, Wiese C (2008). Tailoring a fruit and vegetable intervention on novel motivational constructs: results of a randomized study. Ann Behav Med.

[54] Hawkins RP, Kreuter M, Resnicow K, Fishbein M, Dijkstra A (2008). Understanding tailoring in communicating about health. Health Educ Res.

[55] Wright JL, Sherriff JL, Dhaliwal SS, Mamo JCL (2011). Tailored, iterative, printed dietary feedback is as effective as group education in improving dietary behaviours: results from a randomised control trial in middle-aged adults with cardiovascular risk factors. Int J Behav Nutr Phys Act.

[56] Kreuter MW, Skinner CS (2000). Tailoring: what's in a name?. Health Educ Res.

[57] Brooks R, Rabin R, de Charro F (2013). The measurement and valuation of health status using EQ-5D: a European perspective. Dordrecht: Kluwer Academic Press.

[58] Norman R, Cronin P, Viney R (2013). A pilot discrete choice experiment to explore preferences for EQ-5D-5L health states. Appl Health Econ Health Policy.

[59] Galea S, Tracy M (2007). Participation rates in epidemiologic studies. Ann Epidemiol.

[60] Warner ET, Glasgow RE, Emmons KM, Bennett GG, Askew S, Rosner B, Colditz GA (2013). Recruitment and retention of participants in a pragmatic randomized intervention trial at three community health clinics: results and lessons learned. BMC Public Health.

[61] Méjean C, Szabo DEF, Touvier M, Kesse-Guyot E, Julia C, Andreeva VA, Hercberg S (2014). Motives for participating in a web-based nutrition cohort according to sociodemographic, lifestyle, and health characteristics: the NutriNet-Santé cohort study. J Med Internet Res.

[62] Glanz K, Brug J, van Assema P (1997). Are awareness of dietary fat intake and actual fat consumption associated?--a Dutch-American comparison. Eur J Clin Nutr.

[63] (2007). Australian Bureau of Statistics.

[64] Robertson C, Avenell A, Boachie C, Stewart F, Archibald D, Douglas F, Hoddinott P, van Teijlingen E, Boyers D (2016). Should weight loss and maintenance programmes be designed differently for men? A systematic review of long-term randomised controlled trials presenting data for men and women: The ROMEO project. Obes Res Clin Pract.

[65] Teixeira PJ, Palmeira AL, Vansteenkiste M (2012). The role of self-determination theory and motivational interviewing in behavioral nutrition, physical activity, and health: an introduction to the IJBNPA special series. Int J Behav Nutr Phys Act.

[66] Friederichs SAH, Oenema A, Bolman C, Guyaux J, van Keulen HM, Lechner L (2014). I Move: systematic development of a web-based computer tailored physical activity intervention, based on motivational interviewing and self-determination theory. BMC Public Health.

[67] Miller W, Rollnick S (2002). Motivational Interviewing: Preparing People For Change, 2nd Edition.

[68] Michie S, Abraham C, Eccles MP, Francis JJ, Hardeman W, Johnston M (2011). Strengthening evaluation and implementation by specifying components of behaviour change interventions: a study protocol. Implement Sci.

[69] Alley S, Schoeppe S, Guertler D, Jennings C, Duncan MJ, Vandelanotte C (2016). Interest and preferences for using advanced physical activity tracking devices: results of a national cross-sectional survey. BMJ Open.

