# Correction: Improving Nutrition and Activity Behaviors Using Digital Technology and Tailored Feedback: Protocol for the Tailored Diet and Activity (ToDAy) Randomized Controlled Trial

**DOI:** 10.2196/25940

**Published:** 2020-12-02

**Authors:** Rhiannon E Halse, Charlene L Shoneye, Christina M Pollard, Jonine Jancey, Jane A Scott, Iain S Pratt, Satvinder S Dhaliwal, Richard Norman, Leon M Straker, Carol J Boushey, Edward J Delp, Fengqing Zhu, Amelia J Harray, Maria A Szybiak, Anne Finch, Joanne A McVeigh, Barbara Mullan, Clare E Collins, Syed Aqif Mukhtar, Kieran N Edwards, Janelle D Healy, Deborah A Kerr

**Affiliations:** 1 School of Public Health Curtin University Perth, Western Australia Australia; 2 East Metropolitan Health Service Perth, Western Australia Australia; 3 Cancer Council WA Perth, Western Australia Australia; 4 Health Psychology & Behavioural Medicine Research Group School of Psychology Curtin University Perth, Western Australia Australia; 5 School of Physiotherapy and Exercise Science Curtin University Perth, Western Australia Australia; 6 Cancer Epidemiology Program University of Hawaii Cancer Center Honolulu, HI United States; 7 Department of Nutrition Purdue University West Lafayette, IN United States; 8 School of Electrical and Computer Engineering Purdue University West Lafayette, IN United States; 9 School of Occupational Therapy, Speech Therapy & Social Work Curtin University Perth, Western Australia Australia; 10 Movement Physiology Laboratory University of Witwatersrand Johannesburg South Africa; 11 School of Health Sciences, Faculty of Health and Medicine University of Newcastle Callaghan, New South Wales Australia; 12 Priority Research Centre in Physical Activity and Nutrition University of Newcastle Callaghan, New South Wales Australia; 13 Curtin Institute of Computation Curtin University Perth, Western Australia Australia

In “Improving Nutrition and Activity Behaviors Using Digital Technology and Tailored Feedback: Protocol for the Tailored Diet and Activity (ToDAy) Randomized Controlled Trial” (JMIR Res Protoc 2019;8(2):e12782), corrections have been made to the article title and to three places in the text to reflect that the term “LiveLighter” is a registered trademark.

The original title of this paper was:

Improving Nutrition and Activity Behaviors Using Digital Technology and Tailored Feedback: Protocol for the LiveLighter Tailored Diet and Activity (ToDAy) Randomized Controlled Trial

The title has been changed to:

Improving Nutrition and Activity Behaviors Using Digital Technology and Tailored Feedback: Protocol for the Tailored Diet and Activity (ToDAy) Randomized Controlled Trial

In the originally published paper, the caption of Table 1 read:

Table 1. Frequency of assessment of variables in the LiveLighter ToDAy study for the tailored feedback, active control, and online control groups.

The caption of Table 1 has been changed to read:

Table 1. Frequency of assessment of variables in the ToDAy study for the tailored feedback, active control, and online control groups.

Under the first paragraph of the section "Process Evaluation" in the originally published paper, the sentence:

Selected program completers and noncompleters (tailored feedback and active control groups) will be invited to participate in one-on-one interviews concerning their perceptions of the LiveLighter ToDAy intervention.

has been replaced by the sentence:

Selected program completers and noncompleters (tailored feedback and active control groups) will be invited to participate in one-on-one interviews concerning their perceptions of the ToDAy intervention.

In the originally published paper, the Acknowledgments section read:

Funding for the LiveLighter ToDAy study is provided by a Healthway Health Promotion Research Grant and the East Metropolitan Health Service. The mFR app is funded by NIH-NCI (1U01CA130784-01) and NIH-NIDDK (1R01-DK073711-01A1, 2R56DK073711-04). CC is supported by an NHMRC Senior Research Fellowship and a University of Newcastle (Faculty of Health and Medicine)–Gladys M Brawn Senior Research Fellowship. Funding for Fitabase is provided by a Curtin Institute of Computation grant. The sponsors had no role in the design of the study; collection, analyses, or interpretation of data; writing of the manuscript; and decision to publish the results.

This section has been changed to read:

Funding for the ToDAy study is provided by a Healthway Health Promotion Research Grant and the East Metropolitan Health Service. The mFR app is funded by NIH-NCI (1U01CA130784-01) and NIH-NIDDK (1R01-DK073711-01A1, 2R56DK073711-04). CC is supported by an NHMRC Senior Research Fellowship and a University of Newcastle (Faculty of Health and Medicine)–Gladys M Brawn Senior Research Fellowship. Funding for Fitabase is provided by a Curtin Institute of Computation grant. The sponsors had no role in the design of the study; collection, analyses, or interpretation of data; writing of the manuscript; and decision to publish the results. The term “LiveLighter” is a registered trademark. The LiveLighter campaign is funded by the Western Australian Department of Health and at the time of this study, delivered by the National Heart Foundation (WA Division), and currently delivered by Cancer Council Western Australia.

The correction will appear in the online version of the paper on the JMIR Publications website on December 2, 2020, together with the publication of this correction notice. Because this was made after submission to PubMed, PubMed Central, and other full-text repositories, the corrected article has also been resubmitted to those repositories.

